# Comparison of different assembly and annotation tools on analysis of simulated viral metagenomic communities in the gut

**DOI:** 10.1186/1471-2164-15-37

**Published:** 2014-01-18

**Authors:** Jorge F Vázquez-Castellanos, Rodrigo García-López, Vicente Pérez-Brocal, Miguel Pignatelli, Andrés Moya

**Affiliations:** 1 Fundación para el Fomento de la Investigación Sanitaria y Biomédica de la Comunidad Valencia (FISABIO)-Salud Pública, Avenida de Cataluña 21, 46020 Valencia, Spain; 2 Institut Cavanilles de Biodiversitat i Biologia Evolutiva, (ICBiBE) Universitat de València, Apartado Postal 22085, 46071 Valencia, Spain; 3 CIBER en Epidemiología y Salud Pública (CIBERESP), Madrid, Spain; 4 European Molecular Biology Laboratory, European Bioinformatics Institute, Wellcome Trust Genome Campus, Hinxton, Cambridge CB10 1SD UK

**Keywords:** Viral metagenome, Assembler performance, Taxonomic classification, Chimera identification, Functional annotation

## Abstract

**Background:**

The main limitations in the analysis of viral metagenomes are perhaps the high genetic variability and the lack of information in extant databases. To address these issues, several bioinformatic tools have been specifically designed or adapted for metagenomics by improving read assembly and creating more sensitive methods for homology detection. This study compares the performance of different available assemblers and taxonomic annotation software using simulated viral-metagenomic data.

**Results:**

We simulated two 454 viral metagenomes using genomes from NCBI's RefSeq database based on the list of actual viruses found in previously published metagenomes. Three different assembly strategies, spanning six assemblers, were tested for performance: overlap-layout-consensus algorithms Newbler, Celera and Minimo; de Bruijn graphs algorithms Velvet and MetaVelvet; and read probabilistic model Genovo. The performance of the assemblies was measured by the length of resulting contigs (using N50), the percentage of reads assembled and the overall accuracy when comparing against corresponding reference genomes. Additionally, the number of chimeras per contig and the lowest common ancestor were estimated in order to assess the effect of assembling on taxonomic and functional annotation. The functional classification of the reads was evaluated by counting the reads that correctly matched the functional data previously reported for the original genomes and calculating the number of over-represented functional categories in chimeric contigs. The sensitivity and specificity of tBLASTx, PhymmBL and the k-mer frequencies were measured by accurate predictions when comparing simulated reads against the NCBI Virus genomes RefSeq database.

**Conclusions:**

Assembling improves functional annotation by increasing accurate assignations and decreasing ambiguous hits between viruses and bacteria. However, the success is limited by the chimeric contigs occurring at all taxonomic levels. The assembler and its parameters should be selected based on the focus of each study. Minimo's non-chimeric contigs and Genovo's long contigs excelled in taxonomy assignation and functional annotation, respectively.

tBLASTx stood out as the best approach for taxonomic annotation for virus identification. PhymmBL proved useful in datasets in which no related sequences are present as it uses genomic features that may help identify distant taxa. The k-frequencies underperformed in all viral datasets.

## Background

Metagenomics has been defined as the thorough analysis of the genetic material as directly recovered from environmental samples, including that which is obtained from unculturable organisms [1]. Following the emergence of metagenomics, its quick development responded to the popularization of next-generation platforms. These enabled faster and higher throughput approaches to accurately describe the genetic diversity and elucidate the complex relationships existing between the organisms in different ecological niches. More recently, metagenomics has proven useful for the discovery of new enzymatic functions [2], microorganisms and gene products that may be used for bioremediation [3] and has contributed to the understanding of host-pathogens interactions [4].

The human microbiome has been of special interest in this field, with significant efforts to understand changes in the microbiota or dysbioses that may have an important role in human health and disease [5-9]. The gut is the most densely populated niche in the human body, housing over 10^14^ microorganisms. It has been determined that the core of the intestinal microbiome is constituted by a definite number of nearly ubiquitous species that show a high variability in terms of abundance [8,10] and that this core of species is not shared between close relatives [1,7]. Even though this has been thoroughly explored, as much as 75% of the predicted open reading frames from metagenomic analyses fail to be assigned a function [8].

Most of the previous efforts in metagenomics have been directed towards the survey of prokaryotes and only a few have had bacteriophages and other viruses as their main focus of study. Published viral metagenomes display a low intrapersonal viral diversity and population stability over time but higher levels of interpersonal viral variation [11]. Unlike bacteria, gut bacteriophage populations do not seem to be related between mothers and their twin descendants [12]. The main limitation of working with viral metagenomes is that nearly 80% of the reads yield no significant matches against extant database entries, whereas the remaining 20% are mainly identified as of bacterial origin [11,12]. Such limitations may be explained by the lack of closely related sequences in databases, a common issue with previously unreported viruses and prophages. Since data availability is generally biased towards the most studied human viruses, most databases do not contain enough information to successfully assign identity to the great majority of viral sequence queries from environmental samples. Read length is an additional limitation as reads that are too short often fail to yield functional or taxonomic assignments. As these issues are not restricted to viral metagenomes, microbiome-specific programs have been adapted to address them.

In recent years, several next-generation sequence assemblers have been developed to deal with specific features such as read length, uneven genome coverage values within datasets, efficient managing of computational resources and highly mutational sequence reads. This study focuses on those assemblers that can operate with 454 pyrosequencing data, a technology that has been widely used because of the reads length and sequence coverage, desirable characteristics for *de novo* assemblies and functional annotation [12-21]. Only assemblers previously used or hinted as possible alternatives for viral metagenomic projects have been considered.

Overlap-layout-consensus (OLC) algorithms have proven more efficient for dealing with 454 outputs [22,23]. For this analysis, two of the most popular OLC assemblers were used, Celera and Newbler, which have been extensively used in viral and bacterial shotgun metagenomic projects [8,12,13,16,18,21,24-26] and *in silico* experiments [22-25,27-32]. Additionally, two other OLC assemblers were tested following the authors’ recommendations for working with virus: Minimo, designed for the assembly of small datasets [33] and previously used for virome analyses [12,34]; and VICUNA, an assembler specialized in *de novo* assembly of data from heterogeneous viral populations [35]. Its authors had only used this assembler with single viral populations (e.g. single species).

Assemblers that make use of different algorithms have also been included in this study as alternatives to OLC assemblers. Velvet is one of the most popular de Bruijin graph assemblers [36] and has been used on viral metagenomes using 454 sequence data [15]. MetaVelvet, designed for metagenomic assemblies, is capable of handling different genome coverage values within the different species in the metagenome [37]. Finally, Genovo, an assembler based on generative probabilistic model of read generation, was selected because it uses an iterative algorithm able to estimate the number of genomes in the populations and denoise 454 sequence data [22].

To compare the assemblers, two metagenome datasets were simulated, one composed solely of viral genomes and a second one including prokaryotic and viral genomes (viral-bacterial). Both were based on actual abundance data obtained by Reyes *et al.*[12], using the corresponding organisms' reference genomes, obtained from the Viral and Bacterial NCBI genome database, with the same coverage shown in the actual data.

A critical limitation in viral metagenomes assembly is the lack of a ubiquitous marker, analogous to bacterial 16S rDNA, to identify viral particles and estimate their diversity within ecological niches. Additionally, viral phylogeny based on sequences is impaired by extensive horizontal gene transfer and genome modularity within taxa, which is further complicated by the large numbers of viral particles within environmental samples. This makes it very difficult to find homologous sequences in reference databases. To cope with this, different databases [38,39] and algorithms have been designed which base or complement their taxonomic assignments with genomic features, outperforming pairwise alignment-based approaches such as PhyloPythia [40] and PhymmBL [41]. Other methods that determine taxonomy based solely on k-mer frequencies [42] to improve the sensitivity of taxonomic assignations have not been tested in the context of viral metagenomes. Information on viral communities is still vague and feedback is required for the bioinformatic tools currently in use.

In this work, three approaches for taxonomic assignation were tested. They were selected on the base of their high sensitivity and because they had been used on published viral metagenomic studies:

(1) tBLASTx [43] was chosen because is part of the widely used BLAST suite for sequence alignments. This version enhances the sensitivity to distantly related sequences and has been used widely in viral metagenomic projects [12,14,16,19,44-46].

(2) PhymmBL [41] has been used for bacterial and viral metagenome analyses [18]. It complements sequence alignment information with Interpolated Markov Models (IMMs) based on frequencies of oligonucleotide sequences. This enhances sensitivity without losing specificity. PhymmBL outperforms BLAST predictions when query sequences have no reference in the target database [41].

(3) The distribution of the k-mer frequencies was used to find potential distant phylogenetic relationships. Trifonov and Rabadan [42] proposed a method based on the Kullback–Leibler distance between k-mer frequencies to apply taxonomic assignations using the gamma distribution to assess its significances.

For assessing the functional annotation of viral metagenomes, a third simulated dataset was generated from genomes found in the NCBI Viral genome database to test algorithms that have been used for taxonomic classification in previous works and modified PhymmBL scripts to improve the sensitivity of its taxonomic annotation. The simulations of reads for each of the three metagenomes were carried out using the error rate of 454-pyrosequencing technology.

## Results

### Genome mapping and relative abundance

**Figure 1:**
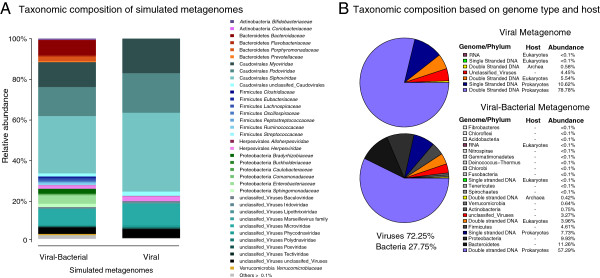
**Taxonomic composition from simulated data.** Taxonomic composition of the simulated metagenomes **(A)**. All families within a phylum or class are represented in a different color gradient. The unclassified virus category refers to reads with no taxonomic information available at class or family levels. Only families representing more than 0.1% of the reads are shown. Taxonomic composition based on genome type and host **(B)**. The upper chart represents the viral simulated metagenomes, while the lower depicts the viral-bacterial metagenomes. For bacteria, phyla are reported instead of genome types. These are shown in a gray-colored scale.

The frequency of each taxon in the simulated metagenomes was estimated to assess how many of the main taxonomic groups of real data were present in the simulated data. The taxonomic frequencies for viruses in the metagenome simulations (Figure 1A) were consistent with those resulting from the taxonomic classification performed by Reyes and collaborators [12]. This was expected since the same taxonomic method, based on tBLASTx [43] with an e-value < 0.001, was used for determining the taxonomic frequencies, and the Viral genome database from the NCBI, used for mapping the reads into reference genomes, shares most entries from the VLP database used by Reyes. In our dataset, the temperate bacteriophages are the most represented type of viral particles (~90% in viral simulation and 65% in viral-bacterial simulation) and a low percentage of eukaryotic viruses (~5%) were found. Our simulations showed that composition was dominated by double-stranded DNA bacteriophages (Figure 1B) from the order *Caudovirales* (families *Podoviridae*, *Myoviridae* and *Siphoviridae*) and single-stranded DNA bacteriophages from the family *Microviridae*. The most representative eukaryotic viruses were double-stranded DNA viruses from the order *Herpesvirales*.

The taxonomic analysis of the viral-bacterial simulation showed that 72.25% of the reads belonged to viral genomes. The reads that were not assigned as viral genomes were compared against the bacterial genomes from the NCBI genome database using SSAHA2 [47]. This fraction displayed the same species distribution present in the viral simulated metagenome. The bacterial species from phyla *Proteobacteria*, *Firmicutes* and *Actinobacteria* were the most represented. These bacteria are the main hosts for the bacteriophages present in the actual metagenome [12].

### Assemblies

Six *de novo* whole-genome shotgun DNA sequence assemblers were tested using the two simulated metagenome based on those by Reyes and collaborators [12]. Several statistics for each assembly were calculated in order to evaluate their assembly quality (the N50, the number of contigs, the largest contig, the number of genomes recovered and percentage of reads assembled) and their contig taxonomic precision (percentage of chimeric contigs, percentage of reads that maps on is original genome, the median value of mapping reads against its original genome, and the number of contigs with an equal percentage of identities between virus and bacterial genomes) in order to estimate the advantages and disadvantages for each assembly (Tables 1 and 2). The N statistics are weighted median statistics reporting the length of the contig in the upper limit of a quantile when all contigs are arranged by size. Thus, 50% of the contigs would be equal to or larger than the N50 whereas 10% of the contigs would be equal to or larger than the N90. Additional values of the N30, N90 statistics are reported in Additional file 1: Table S1.

**Table 1:** Statistics of the viral assemblies

**Assembly**	**Largest contig (bp)**	**N50 (bp)**	**# contigs**	**% chimeric contigs**	**% reads assembled**	**% reads assembled on their original genomes**	**% reads within a viral bacterial hit**	**Genomes recovered**	**% contig identity against its original genome (median)**
Celera 02	15372	1729	5395	15.31	31.06	96.31	0.35	0	98.32
Celera 05	15372	2155	4216	18.43	32.4	95.97	0.35	0	99.09
Celera meta	134824	4816	3165	32.10	52.47	89.80	0.14	1	99.00
Genovo	138337	2357	12258	18.71	98.51	78.73	0.25	0	98.00
Minimo 100/98	1532	744	9763	4.94	3.98	94.83	8.18	0	97.63
Minimo 60/95	2603	471	66769	2.90	37.00	97.41	3.37	0	97.00
Newbler 100/98	78267	1298	4405	20.69	44.41	94.63	0.38	0	99.55
Newbler 60/95	137947	2508	7143	20.84	90.08	93.45	0.23	1	99.00
Velvet	1820	426	10653	4.76	4.96	95.79	6.39	0	97.42
Meta velvet	1820	426	10640	4.71	4.96	95.91	6.41	0	97.00
Optimal	185683	3604	10657	0.00	97.99	99.62	0.24	9	100.00

**Table 2:** Statistics of the viral-bacterial assemblies

**Assembly**	**Largest contig (bp)**	**N50 (bp)**	**# contigs**	**% chimeric contigs**	**% viral-bacterial chimeric contigs**	**% reads assembled**	**% reads assembled on their original genomes**	**% reads within a viral bacterial hit**	**Genomes recovered**	**% contig identity against its original genome (median)**
Celera 02	20633	1646	4932	14.82	0.914	24.95	96.36	0.38	0	98.63
Celera 05	20633	1876	4616	17.20	1.17	26.97	95.88	0.38	0	99.00
Celera meta	134766	2804	4630	30.65	1.99	43.08	88.36	0.17	0	99.00
Genovo	139026	929	33552	29.32	0.55	87.97	84.57	0.21	1	98.00
Minimo 100/98	1636	742	7815	4.84	0.05	3.17	95.03	7.28	0	98.00
Minimo 60/95	3014	518	61092	4.46	0.07	32.10	96.33	3.25	0	97.00
Newbler 100/98	75826	1130	3692	19.97	1.49	30.88	94.69	0.45	0	99.52
Newbler 60/95	137947	1325	10643	20.70	1.15	70.29	93.51	0.27	0	99.10
Velvet	1689	445	18366	9.66	0.19	8.48	91.18	3.68	0	97.56
Meta velvet	1680	445	18663	9.63	0.18	8.47	91.15	3.70	0	97.55
Optimal	153167	1333	27166	0.00	0.00	84.31	99.71	0.32	6	100.00

#### OLC algorithms

They are the most commonly used to assemble 454 reads. Three different implementations were tested: Newbler, Celera and Minimo.

For viral metagenomes, Newbler 60/95 (with parameters of minimum overlap ml = 60 nt and minimum percentage of identities mi = 95%) showed closer results to the optimal assembly. It had the highest percentage of reads assembled (~90%) and a high percentage of reads matching their original genome (see Methods) (~93%), enabling the recovery of a complete genome. However, around 20% contigs were chimeric, a similar result to Newbler 100/98 (with parameters of minimum overlap ml = 100 nt and minimum percentage of identities mi = 98%). Newbler was the most accurate in terms of the identity median against its original genomes, showing the smallest variation across the alignments against the original genomes (Additional file 2: Figure S1). This characteristic makes Newbler a good alternative for genome assembly and recovery in viral shotgun sequence data.

Minimo produced the lowest percentage of chimeric contigs, presenting the strictest assembly (one that limits the number of formed contigs to discard spurious overlaps) in both metagenomes. It had the lowest percentage of reads assembled, the lowest N50 value and the highest percentage of reads within a viral-bacterial hit (Tables 1 and 2). A less stringent version (60/95) resulted in higher percentage of reads assembled and reads matching their original genome, and the largest contig values as well as in lower percentage of reads within a viral-bacterial hit, the percentage of chimeric contigs and N50 values when compared against the most stringent version (100/98). Reducing the collapsing parameters (assemblers’ options that define the threshold for accepting an overlap between two reads) values helps assemble a higher percentage of reads into the assembly with the fewest number of chimeric contigs. Minimo stands as the best candidate to deal with taxonomic annotations.

The Celera assembler had similar results for assemblies 60/95 and 100/98. Its metagenomic parameters improved the percentage of reads assembled and the N50 value, therefore increasing the percentage of chimeric contigs and the reads in their original genome (Tables 1 and 2). Despite the high percentage of chimeric contigs the Celera meta showed highly accurate results in its identity median (Tables 1 and 2) obtaining a low number of large contigs in the viral metagenome simulation. These characteristics are, as well as for Newbler, advantageous for the recovery of entire genomes (Table 1). The main drawback of this assembler is that it reports the highest variation in the percentage of identity (Additional file 2: Figure S1).

As for viral-bacterial metagenomes assemblies (Table 2), there was a decrease in the N50 value, the number of genomes recovered and the percentage of reads assembled. The percentage of similarity within its original genome remained similar, with the exception of Celera, which decreased its variation across the genome mapping despite the higher complexity of the metagenome (Additional file 2: Figure S1, Tables 1 and 2). The percentage of chimeric contigs between viruses and bacteria was insignificant, due to the low effect of the inserted prophages into the bacterial genomes to create chimeras. Newbler again obtained the longest contigs, with more reads assembled and genomes recovered per assembly. Celera with the metagenomic settings produced an assembly similar to that of Newbler with less stringent parameters. Both assemblers maximized the percentage of reads assembled, but also increased the percentage of chimeric contigs (Table 2). Minimo showed similar statistics as the ones in the viral metagenome.

As in other studies [26,30,48] we showed that the stringency of the parameters involving overlapping in both metagenomes influences the quality of the assembly. A decrease in the stringency in the assembly results in the increase of the N50 and the percentage of reads assembled in all assemblers. However, in our analysis, the number of chimeras and misassembled contigs seemed to be independent of these parameters as well as of the percentage of identities. Thus, when overlapping parameters stringency is increased (see Methods), only the contig length and the percentage of reads assembled decrease.

The last OLC assembler that was tested, VICUNA [35], did not produce useful results. When the divergence variable is set to 2% none of the assemblies were able to recover a single contig. By setting it to 10%, the viral assembly yielded six contigs, the maximum number of contigs for VICUNA. The assemblies were not considered for subsequent analyses.

### de Bruijn algorithms

These algorithms, commonly used for short-reads, improve the length of the resulting contigs, therefore minimizing the problem of repetitive sequences [36]. No significant difference between Velvet and its metagenomic version MetaVelvet [37] was found for neither metagenome.

For the viral metagenomes*,* de Bruijn algorithm assemblers exhibited the strictest assembly (Table 1, Table 2), assembling only a small percentage of the reads with the fewest number of chimeras (Table 1). The percentage of reads within a viral-bacterial hit was lower than for assemblies with a similar N50 value such as Minimo 100/98.

Unlike the OLC algorithm assemblers, Velvet and MetaVelvet assembled more reads in complex metagenomes. The N50 and the percentage of reads within a viral-bacterial hit were also increase. The number of chimeras increased with complexity but remained lower than for the OLC strategy (Table 1).

N50 values resulting from de Bruijn assemblers varied depending on the k-mer length but not on the complexity of the metagenome. A scaffolding software may be used in order to increase these low N50 values although it would increase the percentage of chimeric contigs [31].

### Generative probabilistic model of read generation algorithm

Genovo is based on a Chinese-restaurant-process and it can use different coverage values for distinct species within the same metagenomic dataset [22]*.* Genovo uses a generative probabilistic model of read generation, contrary to single sequence reconstruction. This depends on a prior to randomly partition the reads, which is obtained using a Chinese-restaurant-process model. The model is a discrete-time stochastic process generating clusters that accounts for the undetermined number of genomes in the sample. It considers the probability of a read to be assigned to an existing or an empty cluster and continues hill-climbing steps iteratively until convergence is met.

It proved to be the assembler with the best performance in generating long contigs, maximizing the percentage of reads assembled, with a higher value than that expected for the optimal assembly (Tables 1 and 2). However, it generated a large number of chimeras and other misassembled reads (Tables 1 and 2). This may be avoided by increasing the number of iterations. Furthermore, Genovo was time and resource consuming, requiring approximately 3 days for our viral metagenome and up to 21 days for the viral-bacterial metagenome, approximately 80 times more than the second slowest assembler (Minimo).

This assembler drastically increased the percentage of chimeric contigs and the percentage of reads matching their original genome proportionately to the complexity of the metagenome (Tables 1 and 2). Closely related species in the datasets may originate these chimeric contigs.

### Clustering and correlation from assembly parameters

**Figure 2:**
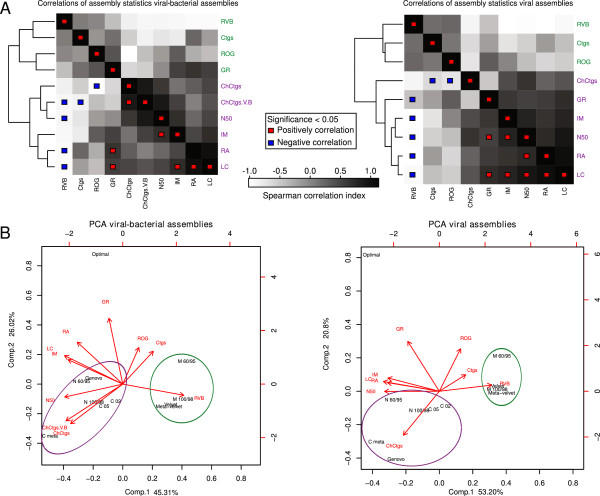
**Correlation analysis from assemblies.** Hierarchical clustering given by the Spearman correlation matrix of the viral-bacterial (left) and the viral (right) assemblies **(A)**. The gradient indicates the strength and direction of the correlations. Blue squares represent significant negative correlations and red squares positive correlations (*P* value ≤ 0.05). Green clusters are based on accurate short-length and low-assembled contigs while purple clusters are based on highly-assembled long-chimeric contigs. Ctgs: number of contigs, ChCtgs: percentage of chimeric contigs, ChCtgs.V.B: percentage of viral-bacterial chimeric contigs, GR: genomes recovered, IM: median of percentage of contig identity against its original genome, LC: Largest contig, N50, RA: percentage of reads assembled, ROG: percentage of reads assembled on their original genomes, RVB: percentage of reads within a viral-bacterial hit. Principal Component Analysis for the different assemblies in both metagenomes **(B)**: The two principal components that better explain variation between assemblies are shown for the viral-bacterial (left) and the viral (right) assemblies. The length of the red vectors represents the effect of each component on the stats. The distance between them indicates their correlation. Circles represent two clusters formed due to the close correlations in the matrices in panel **A**. C: Celera, M: Minimo, N: Newbler, Optimal: optimal assembly.

The Spearman rank correlation coefficient measures the statistical dependence between two variables and creates clusters based on the effect of each assembly statistic on the others (Tables 1 and 2). The correlation gives information about the performance of non-quantifiable variables in real data (number of chimeric contigs, number of reads that assembled into its original genome, the percentage of mapping reads and the percentage of identity against its original genome) using variables that can be measured (number of contigs, N50, percentage of reads assembled and the largest contig). Our results show that the correlation values were lower in the viral-bacterial matrix (Figure 2A), given that its higher complexity reduced the number of reads assembled and the possible observations to create correlations. The hierarchical clustering obtained by the correlation matrix (see Methods) associated the statistics into two clusters based on the contig length and the number of reads assembled (Figure 2B). Cluster 1 is characterized by its positive correlations between the number of contigs, the percentage of reads within a viral-bacterial hit, and the percentage of reads matching their original genome; and by the negative correlations between the largest contig with the N50, the number of reads assembled and the number of chimeric contigs. Conversely, cluster 2 associates the same statistics in the opposite direction. The number of genomes recovered was located in a different group in each metagenome due to its low number of observations. Longer contigs with higher read counts resulted in an increased amount of chimeric assemblies whilst small ones resulted in taxonomic annotations between viruses and bacteria.

### Clustering and correlation from assemblies

The Principal Components Analysis (PCA) was performed using the correlation matrix to understand the effects of the statistics on the assemblies (Figure 2B). For this analysis, we introduced an optimal assembly that maximizes the number of reads assembled into long non-chimeric contigs (see Methods) to determine which assembler or set of parameters had a closer distance to this optimal result.

The PCA from the viral and the viral-bacterial metagenomes show that the optimal assembly is separated from the rest (Figure 2B). Genovo and the OLC Celera with the recommended parameters for metagenomics (Celera meta) and Newbler 60/95 are the ones with more reads assembled, longer contigs and the shortest Euclidean distances, which cluster them with the optimal assembly (Additional file 3: Figure S2). However, they are driven away from the latter in the PCA because of the effects of the percentage of chimeric contigs and the percentage of reads matching their original genome.

The PCA clusters the assemblies into two main groups (Figure 2A). These clusters are seen across both metagenomes with different structure (Figure 2B). One cluster consists of Minimo 60/95, Minimo 100/98, Velvet and MetaVelvet (green circle in Figure 2B) characterized by a large number of short-length contigs, a low percentage of reads assembled, lower percentage of chimeric contigs, and higher percentage of reads within a viral-bacterial hit values. Minimo 60/95 differs slightly from the other elements in this cluster, as it has a higher percentage of reads assembled and a lower percentage of reads within a viral-bacterial hit. A second cluster includes the remaining OLC assemblers and Genovo (purple circle in Figure 2B), driven by the N50, the largest contig, identity median, and the percentage of chimeric contigs. Celera meta and Genovo, in the viral dataset, show the greatest distance from the optimal assembly due to their high the percentage of chimeric contigs.

### Taxonomic analysis of chimeras in assemblies

The taxonomy level at which one or more reads have the same classification is known as the Lowest Common Ancestor (LCA). This was determined for every set of reads within chimeric contigs. For all the assemblers, the LCA tended to be found in lower taxonomic levels (species, genus, subfamily and family), suggesting that chimera formation arises from conserved functions or sequences in closely related species. Neither the complexity of the metagenome nor the collapsing parameters (mi 100/98 ml 60/100 for Minimo and Newbler and utgErrorRate 0.02/0.05 for Celera) noticeably influenced the percentage of chimeric contigs. Instead, it seemed that the contig length, reflected in the N50 value, and the percentage of reads assembled increased the number of chimeric assemblies (Tables 1 and 2 and Figure 2A and B).

**Figure 3:**
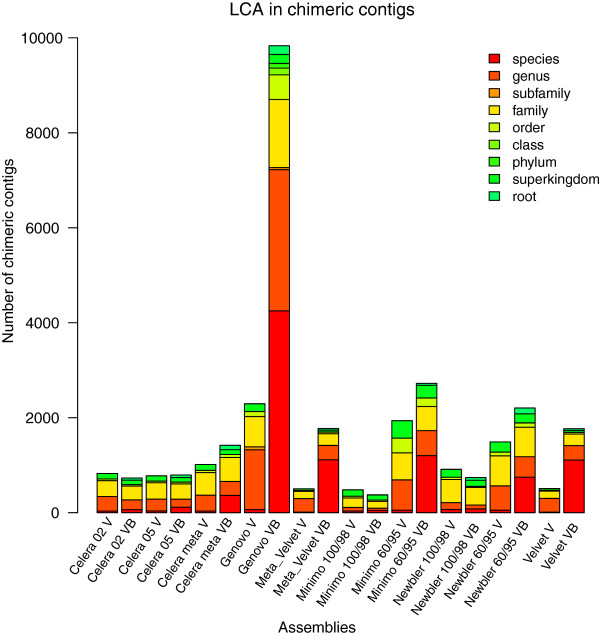
**Lowest Common Ancestor in chimeric contigs.** The LCA of each chimeric contig is represented as a fraction of the total number of chimeric contigs on every viral and viral-bacterial metagenome assembly.

The LCA of the chimeras obtained in our viral-bacterial metagenome assemblies were mainly detected at two taxonomic levels: species (Genovo, Newbler, Minimo 60/95, and the de Bruijn assemblers) and family (Celera assemblies, Newbler 60/95 and Minimo 100/98) (Figure 3). We showed that with less stringent parameters for overlapping (mi 95 ml 60 and utgErrorRate 0.05), the LCA of chimeric contigs tend to be placed at lower taxonomic levels (species, genus, subfamily and family), as seen in the OLC assemblies. Few chimeras were detected between viruses and bacteria in all assemblies (Figure 3).

Chimeras in the viral metagenome mainly occurred at the genus and family levels. The assemblies of viruses had also high percentages of chimeras at the superkingdom (equivalent to domain in the Galaxy output) and order levels. The percentage of higher rank LCA chimeras increased as the overlapping parameters became stricter (mi 98 ml 100 and utgErrorRate 0.02), as seen both for the viral and the viral-bacterial metagenomes (Figure 3).

### Functional analysis of the contigs

The improvement in functional annotations after assembling was tested by contrasting the number and accuracy of the assembled reads annotation against the results from the unassembled reads annotation (Additional file 4: Figure S3). This was achieved by counting the number of times that BLASTx annotation for each read succeeds in assigning the “true annotation” (see Methods section “Functional analysis of the contigs”) to each of the assemblies and the two simulated metagenomes.

Assembling increased the number reads that were assigned a function. The difference between the assembled and unassembled annotations was magnified as the overlapping cut off (the alignment of the best hit against read length) values increased. When the overlapping cut off was set to the 30% of the alignment length, virtually no hits were recovered for the unassembled reads (Additional file 4: Figure S3). This is mainly due to the fact that the unassembled reads do not contain enough information to create an accurate annotation. When the cut off was set below 10%, the differences between the assembled and unassembled functional results were smaller, and in some cases the unassembled recovered more functional hits (Newbler and Celera meta). If the overlapping percentage cut off was increased (see Methods, section “Functional analysis of the contigs”), so was the proportion of correctly annotated functions for most of the assemblers, except for Newbler and Celera meta.

Genovo showed outstanding results in the functional analysis. Unlike the other highly-assembled long-contig assemblies, those obtained by Genovo correctly assigned functions to a higher number of reads, regardless of the overlapping values. Its accuracy was similar to the most stringent assemblies (Minimo and de Bruijn) (Additional file 4: Figure S3).

### Function that causes chimeric assemblies in virus metagenome

**Figure 4:**
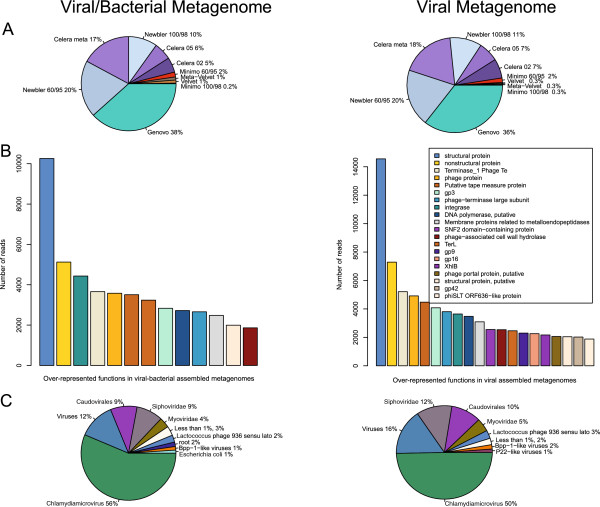
**Functional and taxonomic analyses of chimeric overlaps.** Functional and taxonomic representations for the pairwise chimeric collapses for the assemblers is summarized for the viral-bacterial (left) and for the viral (right) metagenomes. Percentage of chimeric collapses for each assembly **(A)**. Bar chart for the over-represented functions in chimeric read collapses **(B)**. Taxonomy percentage of resulting LCA hits **(C)**.

Horizontal gene transfer within bacteriophages and between them and their bacterial hosts is a common phenomenon [49-53]. In order to determine the number of functions involved in chimeras formation, their taxonomic groups and the assemblies in which they were occur chimeric collapses, BLASTx was used to assign annotations of the chimeric region of the contigs. Assemblies with higher percentage of reads assembled and N50 values accounted for the majority of the events of pairwise alignments between different organisms, hereafter chimeric collapses (detected at least once in every chimeric contig), mainly seen in those from Genovo (Figure 4A).

A total of 1423 different functions were detected to be involved in chimeric collapses in the viral metagenome assemblies with chimeras occurring between different organisms sharing functions. In viral-bacterial assemblies, this number adds up 2656 functions that produce chimeric collapses. The most represented known functions (those with an annotation into the database; see Methods) were taken from both metagenomes assemblies (Figure 4B). The over-represented functions, defined as the outliers from the interquartile range of the frequency distribution of functions involved in chimeric collapses, mainly contained conserved proteins across genomes, such as DNA replication proteins, DNA polymerase, SNF2 domain-containing protein (helicase); proteins involved in DNA packaging such as gp3 terminase, phage-terminase large subunit, Terl, portal proteins gp1 and gp42 [54,55]; hydrolases and lysis proteins such as phage-associated cell wall hydrolase, XhlB and membrane proteins related to metalloendopeptidases (also present in bacterial genomes) [56-58]; DNA transfer proteins such as gp16 [54]; adhesion proteins such as phiSLT ORF636 − like protein [59]; genome integrase [60]; structural proteins, including characterized measure protein [61] and finally unclassified functional proteins.

Most of the chimeric collapses occurred at the genus level (Figure 4C). *Chlamydiamicrovirus* (family *Microviridae*) represents ~50% of all chimeric assemblies for both metagenomes. Genomes from this genus are characterized by their short length. Given this feature and the huge number of reads simulated, the coverage of the genomes was very high and consequently, the number of chimeric collapses tended to increase.

Finally, the low percentage of chimeric collapses observed between bacterial reads may be a consequence of the low coverage for each of the genomes sampled.

### Alternative methods for taxonomic classification

**Figure 5:**
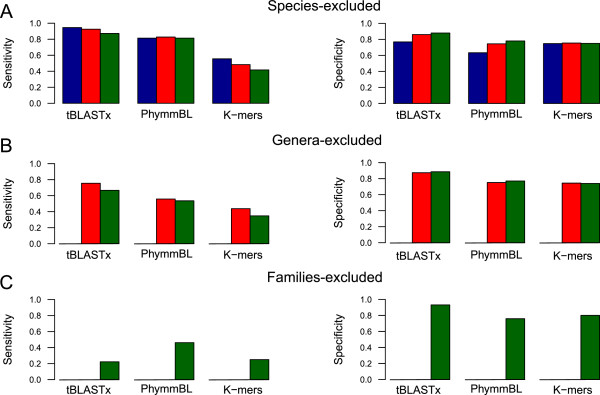
**Sensitivity and specificity of the taxonomic classification programs using custom databases.** The sensitivity and the specificity were calculated based on the results of taxonomic classification trials using three custom databases in which genomes with taxonomic labels matching those in the query dataset were respectively removed: Species-excluded **(A)**, Genera-excluded **(B)** and Families-excluded **(C)**. The query dataset, comprised of the 200 simulated reads, was analysed with three taxonomic classification approaches with each database: K-mer frequencies, tBLASTx, and PhymmBL. These methods were assessed at the taxonomic levels of genus (blue), family (red) and order (green) whenever available for each iteration.

In order to assess the taxonomic classification, their specificity and sensitivity were calculated to standardize the results, based on the correct/incorrect assignations (Figure 5) on a simulated query dataset of 200 reads taken from 19 genomes that were subtracted from the subject database. Three databases were constructed with the remaining genomes, restricting their contents to species, genera or families not included in the query dataset (Species-excluded, Genera-excluded and Families-excluded, respectively). The objective was to assess the performance of different taxonomic classification software when sequences belonging to the same species, genus or family were not found in the database.

The removal of related species, genus or family is reflected by a decrease in the sensitivity for all methods on all taxonomic levels whereas the specificity remains virtually unaltered (Figure 5). The overall proportion of accurate results (the sum of true positives/negatives; mean 77.69 ± 7.27%) is higher than that of incorrect ones (sum of false positives/ negatives; mean 22.31 ± 7.27%), mainly influenced by the number of true negatives and false positives respectively (Additional file 5: Table S2). Within each database analysis, classification at the higher taxonomic levels produces smaller sensitivity values. This is constant with all custom databases.

The frequency of k-mers was the least sensitive of all three methods, with a low number of true positives and a high proportion of true negatives (Additional file 5: Table S2). tBLASTx was the most specific for all taxonomic levels with the three databases and the most sensitive in the Species-excluded and Genera-excluded databases. It yields the highest number of true positives, reporting up to >50% in the order level with the Species-excluded database. The number of false positives it produces is lower than those of the other methods.

As for PhymmBL, we tested for changes in sensitivity and specificity by modifying the options for model structure (separated or combined for each organism) and for alignment method (tBLASTx and BLASTn). From the four different permutations, we selected the one with the confidence score that maximized specificity and sensitivity to compare it with the other taxonomic classification methods (Additional file 6: Figure S4A). The scores that maximized this value at the genus, family and order levels were 0.6, 0.7 and 0.7, respectively. The specificity did not vary significantly between the permutations. The sensitivity of PhymmBL using tBLASTx was higher than with BLASTn. The former was selected as the alignment method along with single models for the rest of the analysis with this program.

PhymmBL produces similar results to those of tBLASTx, but gets a lower proportion of true positives and a higher number of false positives resulting in a decrease in sensitivity and specificity in all taxonomic levels, except for the Family-excluded database, in which PhymmBL obtains higher sensibility values.

## Discussion

The performance of several read assembly and gene annotation tools has been tested to study simulated viral and viral-bacterial metagenomes. Taking into account the lack of information in the current databases we tested the impact of the assembly process on the accuracy of the taxonomic and functional annotations [62-64].

Even though simulated metagenomic datasets are an oversimplified emulation of actual metagenomic data, the conclusions drawn from these analyses are still valid as the focus of this study was to assess the performance of different assemblers rather than recovering the exact underlying taxonomic distribution of the data.

Viral metagenomic assemblies have been classified into two groups. The first one is characterized by their low percentage of chimeric contigs, high prevalence of the reads within a viral-bacterial hit and short contigs while the second is defined by their high percentage of reads assembled, as well as long and chimeric contigs. For both types of assemblies the percentage of reads matching their original genome was high, with similar identity median.

The OLC-algorithm assemblers show a wider spectrum of results, requiring less time and computational resources, making them more suitable when dealing with highly heterogeneous metagenomes. This allows the user to choose between two types of assemblies: those with many accurate micro-contigs, at the expense of the capacity of taxonomic/functional prediction, and those with longer contigs, enabling the recovery of whole genomes or more taxonomic functions.

Newbler and Celera, the less strict assemblers, produce the largest OLC-assemblies, with the highest percentage of reads assembled, and increase the probability of reconstructing whole genomes. In contrast, Minimo, the most conservative algorithm, shows a better accuracy but the percentage of reads assembled and N50 values are reduced, while increasing the percentage of reads within a viral-bacterial hit. Interestingly, the performance of Minimo 60/95 is positioned between that of Newbler/Celera meta and Minimo 100/98. It assembles significantly higher proportions of reads than its most stringent version, with the same accuracy whilst reducing the number of reads within a viral-bacterial hit. For all of these assemblies the identity median and, except for Celera meta, the percentage of reads matching their original genome values are similar.

The stringency of the parameters for length of overlap and minimum percentage of identity does not significantly increase the quality of the contigs or the accuracy in functional annotation (except for Celera meta). Stringent parameters were expected to reduce the number of chimeras, but correlation analysis of the assembly statistics show that they result in a reduction of the percentage of reads assembled and N50 values, whereas the percentage of chimeric contigs remained virtually unaltered. Assemblies with restrictive parameters have more LCA hits resolved at phylum, superkingdom and root taxonomic levels. This may be explained by the fact that only conserved sequences across genomes have a percentage of identity that leads to the formation of chimeric assemblies. Some of these proteins are ubiquitous in the microbiome such as housekeeping genes and genes involved in phage structure or replication (Figure 4B). Higher complexity is associated with lower N50 and the percentage of reads assembled values, as would be expected in lower species-level coverage scenarios.

An increased N50 value results in a higher number of predicted functions but the percentage of correct assignations decreases. This effect is drastically observed in Newbler and Celera meta, which obtain around 40-50% of correctly annotated functions. For the recovery of long contigs, including whole genomes, Newbler provides the best results, with higher accuracy than Celera meta.

Minimo 60/95 generates roughly the same number of correctly annotated reads but with much higher accuracy. Although the N50 value in Minimo is 10 times lower than that of the optimal assembly, the results suggest that it could be the assembler of choice for diversity analysis of viral metagenomes obtained with 454 technology provided that less stringent parameters are selected (60/95 in this study).

VICUNA [35], failed to achieve an acceptable assembly for comparison. This outcome was not unexpected as it had been used for a single viral population per run (e.g.: an HIV population) and had just been suggested, but not tested, for metagenomics.

Previously filtering the reads to separate specific genomes using a mapper such as SMALT [65] (Ssaha2’s virtual successor), would enable the usage of VICUNA. The mapper could be used as a first approach along with the viral database to allocate the reads into possible genomes, classifying each set and assembling them separately. Still, this would not be equivalent to other of the assemblies and was consequently discarded from the rest of the analysis.

Velvet and MetaVelvet, de Bruijn graphs assemblers, are conservative, ensuring good quality contigs and highly reliable functional annotations. However, the resulting contigs, which have short N50 and low percentage of reads assembled, may be used for successive rounds of assembly or scaffolding. Unlike the percentage of reads assembled, the N50 seems to be independent of the assembler and the complexity of the metagenome. Our results showed that de Bruijn assemblers cannot handle 454 sequences optimally, as they generate short contigs and show a low percentage of reads assembled. These results are further supported by other studies [22,23].

The generative probabilistic model of read generation algorithm assembler, Genovo [22] can deal with high levels of taxonomic heterogeneity, because it can input a different coverage for different contigs rendering it more sensitive to underrepresented species. Genovo shows the highest N50 with the largest contig, assembling most of the dataset into the viral metagenome. Horizontal gene transfer, the low number of different functions across bacteriophages, and the effects of multiple coverage estimations lead Genovo into successfully merging chimeric contigs that share common functions, rather than reflecting their actual taxonomy. This feature makes Genovo the assembler of choice for functional annotation due to its high number of functional assignations and their accuracy.

The high percentage of chimeric contigs may be caused by the clustering of related functions from different organisms. This is consistent with the fact that there are so many chimeras at order, class and phylum taxonomic levels. For most of the assemblies, the number of reads with a correct functional annotation is higher in the contigs than in the original unassembled dataset. If the overlapping percentage value is increased (see Methods), this effect is magnified. The number of predicted functions from unassembled reads decreases abruptly at higher overlapping percentages, virtually disappearing beyond 50% (Additional file 6: Figure S4A).

Bacteriophage integration into genomes (prophages) appears to have just a limited influence in the formation of chimeras, most likely because those occurring between viruses and bacteria are mainly determined by the contig length, just like the rest of the chimeric events, and are practically non-existent in the assemblies.

As the percentage of reads assembled decreases, so does the contig length, the number of chimeras and the percentage of reads matching their original genome, whereas the percentage of reads within a viral-bacterial hit increases. Thus, shorter reads are also less useful for taxonomic and functional profiling. This happens both in the viral and the viral-bacterial metagenomes, meaning that the effect of prophages may arise from a taxonomic assignation bias rather than from an aberrant assembly. Increasing the contig length can improve the accuracy when dealing with viral-bacterial metagenomes, despite the chimeric contigs between both. Furthermore, the results may be improved by effectively selecting the viral hits rather than the bacterial ones when e-values are identical.

Most LCAs in chimeric contigs are detected in the class, order and phylum taxonomic levels. This could be explained by the inconsistent levels in viral taxonomy and the high number of functions that are shared between viral genomes.

Determining the LCA of the assembled contigs can support the taxonomic level assignation at which annotations should be given.

Most functions are detected in chimeric alignments. The most over-represented functions are basic for viral replication and are conserved across all viruses (Figure 4). This may be attributed to different causes such as horizontal gene transfer according to the modular theory of bacteriophage evolution in which bacteriophages are considered a group of interchangeable genetic elements [66-68]. Furthermore, the combination of closely related species with small genomes and a high prevalence in a niche often leads to the occurrence of chimeric collapses in the assemblies as seen in the genus *Chlamydiamicrovirus*.

Assembling provides a useful platform for taxonomic and functional analyses of viral metagenomic datasets but this is also extended to diversity analyses which can be measured using programs such as PHACCS [69] and CatchAll [34], which take into account the contig spectrum to measure the overall diversity within samples.

With respect to the comparison between different taxonomic assignment approaches, we suggest they may be combined to obtain a better result, although this approach was not fully tested for this study. Because of the intrinsic differences of the algorithms, each one provides an advantage in different applications and may be used complementarily.

As for the second part of the analysis, the methods for taxonomic classification are mostly conservative as they show a high proportion of true negatives, low numbers of true positives and high specificity scores for all the analyses. Values are consistent with previous studies [16,18,42] and support all three programs as reliable alternatives for viral taxonomic assignation in different specific scenarios, even though the total true positives are limited. This number is further affected by the removal of the sequences from the databases.

The frequencies of k-mers within the genomes were not very sensitive with any of the databases and taxonomic levels. However, this method presents the highest numbers of true negatives in most analyses. Although it is not the aim of this study, the k-mer approach may potentially be used to differentiate bacterial from viral sequences so that the set of reads may be cleansed. This method stands out because it does not depend on alignments between the databases and can thus detect sequence homology in relatively distant genomes.

tBLASTx is a good taxonomic assignation approach for viral metagenomes, displaying higher sensibility and the specificity scores of PhymmBL and k-mer frequencies in most cases. It is highly specific because it produces a high number of true positives and few false positives. Because of the low number of available viral genomes this program may be limited to extant sequences in the databases and does not retrieve information from other genomic features unlike the other two methods. This limitation is reflected by the loss of sensibility with the Families-excluded database.

PhymmBL yields results that fairly resemble those of tBLASTx. However, it stands out as the most sensitive program for viral taxonomic assignation when no genera or families match those in the query dataset (Families-excluded database). The algorithm can be enhanced by selecting tBLASTx instead of the default BLASTn. Even if no significant alignment is possible, the program can resolve the taxonomy of any query using IMMs. Nevertheless, PhymmBL is computationally demanding and its code was developed for usage with bacterial datasets, as input database structure is expected to contain all taxonomic levels used in bacteria.

When the query dataset is analysed using all three different programs with the Species-excluded and Genera-excluded databases, it is shown that lower sensitivity scores are obtained in higher order taxonomic levels, namely in the order and family levels. These may seem counterintuitive, since the exact opposite is seen in bacteria, and may be due to the broad genetic heterogeneity in viruses [70]. MAP (Metagenomic Assembly Program) [28] and Ray Meta [71] (a short read de Bruijn assembler) came out around the time when this work was being carried out. MAP (a metagenomic OLC assembler) is expected to improve OLC single genome assembly by taking into account mate-pair information for the lay-out stage [28]. Our datasets were modelled after 454-pyrosequencing data and included no mate-pair information. A new customized dataset especially designed for MAP would have been required. This would render direct comparison impossible. Ray-meta may be one of the best NGS assemblers for short-read sequence data. However, as stated above, the results from this and other studies suggest that de Bruijn assemblers are not an optimal approach. Furthermore, we tried to limit the study to those assemblers that had at least been tried for viral genomics. Therefore, we decided not to include them in the analysis.

## Conclusions

In this work, we measured the effect that assembling simulated viral gut metagenomes with different assemblers had on the quality of taxonomic and functional annotations. None of the assemblers managed to generate results that truthfully resemble the optimally assembled metagenomes.

The success of most assemblies is greatly hindered by the formation of chimeric contigs. As supported by our data, chimeras are ubiquitous in all assemblies. They are formed at virtually any taxonomic level or function, regardless of the stringency of the parameters and the existence of reads of bacterial origin in the dataset.

Depending on the objective of each project, we propose two ways to assemble 454 sequence data from viral metagenomic data. Diversity and taxonomic analysis may benefit from using Minimo with ml60 and mi95 parameters as it minimizes the number of chimeric contigs with an acceptable percentage of reads assembled. On the other hand, Genovo stands out in functional annotation analyses, as it forms the longest contigs and has the highest percentage of reads assembled. Since Genovo is time and resource consuming, Newbler can be considered as a cost-efficient alternative.

Additionally, different taxonomic assignment programs were tested to evaluate specificity and sensitivity of taxonomic assignations as well as the effect of removing sequences that were close to the query dataset in different taxonomic levels. Methods vary in terms of assignation success, with tBLASTx as the most successful and accurate in most cases. The frequency of k-mers was the method that yielded the lower overall scores for virus analysis. Because of the intrinsic differences of the algorithms, each one provides an advantage in different applications and may be used complementarily, although this approach was not fully tested for this study. To make the most out of them the k-mers frequency method could be used to separate bacterial or specific subtypes of viral particles, tBLASTx, due to its specificity and sensitivity, would be a good option as the main taxonomic classification program and, PhymmBL due to its sensitivity could be a good choice to obtain information where others cannot, especially if the available reference database lacks closely related species.

## Methods

### Collection and processing of viral metagenome sequences

Virus-like particles sequences deposited with the accession number [SRA:SRA020605**]** by Reyes, *et al.*, [12] were downloaded from GenBank/EMBL/DDBJ Short Read Archive. The fastq-dump.2.1.18 program from SRA toolkit was run to generate the FASTQ sequences from all the SRA files. PRINSEQ (lite 0.14.4) [72] (−derep 1 -min_qual_mean 20 -ns_max_n 1), was used to remove redundant reads and trim low-quality reads. Tagcleaner (−predict -mm3 1) [73] was used to remove tags.

### Genome mapping and coverage calculation

In order to find their taxonomic identity, all cleansed reads from the original virus-like particles metagenomes were mapped against all bacterial and viral reference genomes from the NCBI genome database (May 2012). Reads were assigned to genomes if their alignment against the reference genome comprised at least the 60% of their length. Given its high variability and the low number of viral genomes, viral mappings were carried out with tBLASTx (e-value < 0.001) [12,16] against the viral reference genome database. This compares the protein translation of the six reading frames of a query DNA sequence against the translated sequence of the six frames of a target, thus enhancing the sensitivity to distant relationships in DNA sequences. All reads assigned to viral genomes were excluded from the bacterial mapping. The remaining reads were aligned using SSAHA2 software [47] against the bacterial reference genome database, as this is a best approach for genome mapping. The best hit (that with the highest percentage of alignment) from each read was used for a particular genome coverage calculation.

The coverage for each genome was calculated using the following formula:

$$\mathrm{Coverage}G_{i}=\sum L_{\mathrm{ri}}/G_{i}L$$

Where *L*_*ri*_ is the length of every read that matches the *i*^*th*^ genome (*G*_*i*_) and *G*_*i*_*L* is the length of the *i*^*th*^ genome. The final coverage of every genome was estimated as the mean of all values obtained from each metagenome.

### Metagenome simulations

The genome coverage values were standardized by adjusting them to the lowest value. These were used to generate the frequencies in a .mprf file, used by the program MetaSim [74] to obtain the relative abundances and simulated its proportional number of reads. Two different sequence datasets were simulated: the first one contained 579 viral genomes and the second one a mixture of 1152 viral and bacterial genomes (Figure 1). Except for the genome frequencies and composition, the same simulation settings were used for both metagenome simulations, taking into account the 454 error model [75]. A total of 500000 reads with an average length of ~400 bp were simulated (Table 3). Each read was assigned the taxonomic information of its source genome as well as its position in the chromosome. The header for each simulated read contained the taxonomic id, the position of the errors added, the genome from which it was simulated and the position of the read into the genome; this information was required to reassembled the original genomes form the simulated data and to assess the accuracy of the assemblers.

**Table 3:** Characteristics of the simulated data

	**Genomes**	**# Reads**	**Average read size**	**# Base pairs**
**Viruses**	579	500000	434.26 pb	217130409
**Viruses-Bacteria**	1152	500000	431.11 pb	215556023

The metagenome simulations *mprf* files that contain the relative abundance for the simulated metagenomes are available into the Additional file 7: Dataset S1. The genomes names and the number of read calculated for each of them are available into the Additional file 8: Table S3 for the Viral-Bacterial metagenome and the Additional file 9: Table S4 for the viral metagenome.

### Assembly

Contigs longer than 350 bp were taken into account to perform the data analysis.

The following assemblers were used:

1) *Newbler* is the default recommendation for assembling 454 reads (Roche) and has been used for viral analyses [12,14,16,18,21]. The parameters were set to minimum length of overlap (ml) = 60/100 and the minimum percentage of identical base pairs (mi) = 95/98 as used in published works [12,14,16,30]. Additionally, the option –ace was used to obtain a map that would allow read tracking within the assemblies and parameter -a 350 to avoid reads below such contig length. Several other parameters have been tested in other works, (ml = 40 and mi =85/90) but they do not seem to affect the assembling of 454 reads [30].

2) *Celera*[76] has been extensively used for metagenomics. Contig error rate (utgErrorRate) was set to 0.02/0.05 in order to make the results equivalent to the other OLC assemblers (Newbler and Minimo). Just as Newbler, Celera assembler has been used in viral metagenomic analysis [18] and several metagenomic projects [26,48,63,77]. An additional iteration with utgErrorRate = 0.12, as recommended for metagenomics by Rusch *et al.* (2007) [48] and genome size (utgGenomeSize) = 1/50 of the total number of bases, an artificially small value to avoid a high penalty to the assembly caused by the variable coverage between species [26].

3) *Minimo*[33] was designed to assemble small datasets and has been used for virome analyses[12,34]. In order to make the results equivalent to those from other OLC assemblers, the parameters were set to a minimum contig overlap length MIN_LEN = (60/100) and a minimum contig overlap identity percentage MIN_IDENT = (95/98). The program was also executed with options FASTA_EXP = 1 to input fasta format files and -D ACE_EXP = 1 to obtain a map to locate the reads within the assemblies.

4) *Velvet*[36] has a good performance with short-read datasets and has been used in viral 454 sequence metagenomic projects [15]. It yields highly reliable contigs and it is not so affected by repeated areas between contigs. Velveth and velvetg modules were run with different k-mer lengths (15–49 bp). Assemblies with the highest N50 values and the maximum percentage of reads assembled were considered the best ones. For the final assembly, velveth was run with a k-mer size of 49 and the -fasta –short options; velvetg was run with options -read trkg yes -amos_file yes in order to create a traceable map of the graph of each contig and a min read length of 350 bp -min_contig_lgth 350.

5) *MetaVelvet*[37] is the metagenomic version of Velvet. It can be given multiple coverage values in the form of an array using the option exp_cov_multi; the spectrum of genome coverage values is given after each run of meta-velveth and meta-velvetg; each assembly must be run twice, first to obtain the list of coverages and second to use this list within the assembly. The remaining settings were the same as the ones used for Velvet.

6) *Genovo*[22] uses a probabilistic model that calculates different coverage values to assemble metagenomes. It improves gene recovery and gathers more reads than single-genome assemblers. A hundred iterations were run to improve the assembly. The program BLAT [78] was used to assign each single read to a contig and create an ACE-like output format. It was assumed that a read must belong just to a single contig, its alignment must be over 60% of its length and should present at least 95% of sequence identity. Customized Perl scripts were developed to parse the output and create a minimum contig length of 350 bp.

7) *Vicuna *[35]: VICUNA is an assembler designed for genetically heterogeneous populations such as viral ones. The assembler was run using *de novo* whole genome sequences [35]. The native input for the assembler are paired-end sequences, so we used the script fakePairedReads.pl, available into the VICUNA package, to create an artificially paired-end reads input dataset. All assemblies were run with default options with the exception of the -min_output_contig_len 350 to obtain contigs longer than 350 bp and three different values for the Divergence variable (2, 5 and 10). The variable MSAFileName was not defined so no fasta files storing Multiple Sequence Alignment were used to assembled reads into contigs.

All assemblies were run with options that allowed for read tracking.

### Assembly evaluation

Perl scripts were developed to extract the information contained in the selected output file format, ACE, to calculate the longest contig, the percentage of reads for each assembly and the N statistics values, defined as the contig length in which the summation of the total bases in the contigs arranged by size accounts for 90%, 50% and 30% of the nucleotides of the whole assembly. The percentage of chimeric contigs was determined by checking the taxonomic information of every read that composed each contig. Whenever a contig included reads from different organisms, the contig would be considered a chimeric contig. The NCBI’s “Gi” sequence identifier of each of our simulated reads was used to obtain its complete taxonomy with the Fetch taxonomic representation tool (version 1.1.0) in the Galaxy server [79]. For each of the chimeric contigs, the level of the LCA was determined as the taxonomic level in which all the reads possess the same taxonomic annotation.

### Taxonomic classification

The taxonomy of each contig was assigned after that from the organism with more reads within the contig. In order to measure the accuracy of the contigs, Megablast [80] was used with default parameters to map them against the viral and bacterial reference genomes from the NCBI database. It was preferred over SSAHA2 because the latter is limited to similar sequence searches, potentially ignoring true annotations in a highly heterogeneous database (virus and bacteria). Additionally, megablast can deal better with divergence mappings and parallelization, and it is less computationally demanding. The high-scoring segment pairs (HSP) were obtained for each contig and the percentage of identity against its original genome was calculated as the ‘contig score’ [31]. This score is calculated by adjusting the percentage of the contig in the HSP to the percentage of the identities in the alignment. The percentage of correctly annotated reads was calculated as the number of reads that matched the taxonomy of their genome HSP.

Within contigs with correctly assigned taxonomy, the number of reads that have a matching taxonomy with that organism was counted. The resulting sum was divided by the total number of reads assembled to obtain the percentage of reads matching their original genome.

Percentage of reads in the assembly that are correctly assigned to the organism.

If a contig yielded at least one viral and one bacterial hit, both with the same contig score, this contig was counted as a viral-bacterial hit. The sum of all these reads within the contigs was divided by the total number of reads in the assembly in order to calculate the percentage of reads within a viral-bacterial hit.

Whenever an alignment was formed between the contig and a reference genome and it spanned 100% of its length whilst identity remained above 95%, that contig was considered as a whole genome assembly.

### Optimal assembly

An optimal assembly, defined as the assembly that best resembles the original genomes, was obtained for the viral and viral-bacterial simulated metagenomes. These were generated based on real genomic data coordinates obtained for each read from the output of the MetaSim software. A Perl script was created to use this information, along with the original reference genomes, to generate a map of coordinates containing the numbers of the first and last bases of each read with respect to its original genome. This map was then used by the script to assemble contigs that fitted the original genomic sequences without any artefacts that may have been introduced by the assemblers. All contigs were analysed following the same pipeline that was used to measure the quality of the assemblies.

### Cluster and correlation analyses of the assembly statistics

The R (2.12.0) package [81] was employed to perform the correlation analysis of the assembly statistics from the simulated datasets (Tables 1 and 2). PCA were performed with the *princomp* function (scores = TRUE, cor = TRUE). The correlation matrix was calculated based on the Spearman's *rho* statistic, *cor* function (method = spearman). The assembly stats were hierarchically clustered, based on the euclidean distance from their results, using the *hclust* function (method = complete) (Figure 2A). The same methodology was applied to the analysis of the correlations between statistics and assemblies (Additional file 3: Figure S2).

### Functional analysis of the contigs

In order to standardize functional annotations from the viral-bacterial metagenome database, protein sequence files (FAA) located into the NCBI whole genome database (May 2012) were downloaded. All proteins were compared with a custom concatenated database composed of eggNOG [82], ACLAME [83] and POG [38] (May 2012). BLASTP [84] was used (e-value < 0.000001; alignment > 70%) to determine the identity of each protein sequence, based on the best match against the database (lower e-value and highest percentage of identity). The coordinates of each open reading frame were retrieved from the PTT files (May 2012). The position of every read in the reference genome was used to determine its NOG, POG or ACLAME functional classification, considering it to be its “real annotation”, provided that at least a 60% of the read was aligned.

The contigs from the different assemblies were compared with the same custom database using BLASTx [85]. Due to their augmented length, sequences within contigs may span several open reading frames, resulting in several non-overlapping functional assignations taking the best hit in each region (e-value < 0.000001; alignment > 70%) as its function. For each contig, all reads within the annotated regions inherited the same functional classification determined for that region. Likewise, the two unassembled metagenomes were analysed using the same methodology.

In order to compare whether read assembling affects functional annotation, the functions assigned to assembled reads were compared against the ones reported for the same set of unassembled reads using different cut off levels in the overlapping percentage, calculated as the length of the alignment with the best hit divided by the total read length (Additional file 4: Figure S3).

### Functional analysis in chimeric contigs

The taxonomic information was used to compare the LCA for each pair of reads that overlap within contigs. The number of times that each LCA was reported above the species level was counted, as well as its functional annotation and the assembler from which the contig originated.

Because of the high number of chimeric functions, the *boxplot* function (plot = FALSE) from the R package was used to recover all the outlier functions. Only the outlier functions with abundance > 1% were plotted into the bar charts (Figure 4B).

### Analysis of taxonomic classification methods

A total of 19 genomes were randomly picked from the viral reference genomes from the NCBI genome database (May 2012) to simulate 200 reads, a query dataset, with the same parameters used for both metagenome simulations. The remaining genomes were used to construct three different custom databases for measuring the performance of the taxonomic assignment methods when dealing with unknown viral-like particles at specific taxonomic levels: i) Species-excluded database, in which all the genomes bearing the same species as the query dataset were removed. ii) Genera-excluded database, in which sequences with the same genus as query dataset were removed. iii) Families-excluded level, in which all genomes with families matching those in the query dataset were removed. This was carried out in order to simulate different databases in which no closely related species, genera or families were present.

Each of the results was compared at the different taxonomic levels that were available in every case (order, family and genus levels for the Species-excluded database, order and family using the Genera-excluded database, and order with the Families-excluded database).

The taxonomic assignation was carried out using the following programs:

1) *PhymmBL*[41] was optimized using the *Combine files* boolean option in two separated iterations to create both joint and separate IMMs. Two different Blast algorithms (BLASTn and tBLASTx [43]) were tested to determine the settings that maximized sensitivity and specificity (Additional file 6: Figure S4). The IMM models and BLAST database required by PhymmBL were constructed using the "customGenomicData.pl" script provided in the PhymmBL package using the taxonomic information obtained from the Galaxy genome server. Cut off values for confidence scores were set to 0.6, 0.7 and 0.7 for the genus, family and order levels respectively in order to maximize sensitivity and specificity.

2) *K-mers frequency.* These frequencies were analysed according to Trifonov and Rabadan (2010) [42]. The *Oligonucleotide Frequency* function, contained in the Biostrings library [86] of the R package, was used to obtain the k-mers frequencies for all sequences and genomes. The resulting frequency matrix was analysed with the *Kldiv* function to calculate the difference between frequencies based on their Kullback–Leibler distance. Reads were annotated to a genome if the distance between them was maximized and its Z value, taken from a gamma distribution [42], was > 0.05.

3) *tBLASTx.* The 200 reads were compared with the control database, taking the best hit as the read annotation (e-value < 0.001) as seen in [12,16,17].

### Specificity and sensitivity

We calculated the number of true positives (TP), true negatives (TN), false positives (FP) and false negatives (FN) for each of the classification methods at two taxonomic levels: genus and family. The specificity and sensitivity were determined using the following formulas:

$$\mathrm{sensivity}=\frac{\mathrm{TP}}{\mathrm{TP}+\mathrm{FN}}$$

$$\mathrm{specificity}=\frac{\mathrm{TN}}{\mathrm{TN}+\mathrm{FP}}$$

## Abbreviations

HSP: High-scoring segment pairs; IMM: Interpolated Markov Model; LCA: Lowest Common Ancestor; MAP: Metagenomic Assembly Program; OLC: Overlap Layout Consensus.

## Competing interests

The authors declare that they have no competing interests.

## Authors’ contributions

JFVC, MP and AM conceived the experimental design. JFVC performed computational analysis, interpreted and discussed the results and wrote the first draft of the manuscript. RGL and VPB participated in the discussion and co-wrote the manuscript. AM provided funding and computational resources. All authors read and approved the final manuscript.

## Supplementary Material

Additional file 1: Table S1. N Statistics. Table including the N30, N50 and N90 statistics for each assembly using the virus and virus-bacteria datasets. Click here for file

Additional file 2: Figure S1. Graphical representation of the percentage of identity variation. Boxplots contrasting percentages of contig identities against their original genomes as seen in viral **(A)** and viral-bacterial metagenome simulations **(B**). Outliers are not shown. Click here for file

Additional file 3: Figure S2. Hierarchical clustering from Spearman correlation coefficient. Heatmaps representing the parameter correlations in viral **(A)** and viral-bacterial assemblies **(B**). Two main clusters (green/purple) are shown between assemblies associated with their assembly statistics. Black squares indicate positive correlations and white squares negative ones. Ctgs: number of contigs, ChCtgs: percentage of chimeric contigs, GR: genomes recovered, IM: median of percentage of contig identity against its original genome, LC: Largest contig, N50, RA: percentage of reads assembled, ROG: percentage of reads assembled on their original genomes, RVB: percentage of reads within a viral-bacterial hit. Click here for file

Additional file 4: Figure S3. Contig Functional Annotation. Functional annotation of the assembled reads in the viral-bacterial **(A)** and viral **(B)** metagenomes. Bar charts summarize the number of reads with correct (blue) and incorrect (red) assignations at different levels of overlapping cut off values (>10%, >30%, >50% and 100%). Dark colors are used for unassembled reads and light colors for assembled ones. Boxplots show the percentage of correct annotations (right side of A and B), considering all assemblies at four different cut offs for overlapping percentages. Boxplots per assembly (down) indicate the percentage of the correctly assembled reads for each assembly at 10 different cut off intervals (10% to 100%). Click here for file

Additional file 5: Table S2. Sensitivity and Specificity Statistics. True positives, false positives, true negatives and false negatives were calculated based on the results of taxonomic classification trials using the three custom databases Species-excluded, Genera-excluded and Families-excluded at the available taxonomic levels. The % Correct Annotation column was calculated as the sum of the true positives and true negatives. The % Incorrect Annotations is calculated as the sum of the false positives and false negatives. Click here for file

Additional file 6: Figure S4. PhymmBL model and alignment method comparison. Sensitivity and specificity were compared at the genus **(A)**, family **(B)** and order** (C)** taxonomic levels. Different iterations selected all available permutations using single models (Sm) or mixed models (Mm) and alignment using BLASTn (Bn) or tBLASTx (tBx) for the Species-excluded database. Click here for file

Additional file 7: Dataset S1. Simulated dataset files. Files with *mprf* extension contain the relative abundance data used for simulated metagenomes. They are the input for the MetaSim program. Files with *msim* extension summarize the data after simulations. They are the output from MetaSim. Click here for file

Additional file 8: Table S3. Viral-Bacterial genomes frequencies. Table including the NCBI Accession, Gi, the species and the number of reads for each of the genomes selected for the viral-bacterial metagenome simulation. Click here for file

Additional file 9: Table S4. Viral genomes frequencies. Table including the NCBI Accession, Gi, the species and the number of reads for each of the genomes selected for the viral metagenome simulation. Click here for file
