# Supplementary material for: Comparison of different assembly and annotation tools on analysis of simulated viral metagenomic communities in the gut
Source: BMC Genomics. 2014 Jan 18;15:37. doi: 10.1186/1471-2164-15-37 (PMC3901335; doi:10.1186/1471-2164-15-37)
Supplement: Additional file 1: Table S1. — N Statistics. Table including the N30, N50 and N90 statistics for each assembly using the virus and virus-bacteria datasets. [file 1471-2164-15-37-S1.docx]

| Dataset | Assembly | N30 | N50 | N90 |
| --- | --- | --- | --- | --- |
| Virus |  |  |  |  |
|  | Celera 02 | 2740 | 1729 | 1071 |
|  | Celera 05 | 3229 | 2155 | 1140 |
|  | Celera meta | 18099 | 4816 | 1290 |
|  | Genovo | 10512 | 2357 | 628 |
|  | Meta Velvet | 517 | 426 | 361 |
|  | Minimo 100/98 | 778 | 744 | 657 |
|  | Minimo 60/95 | 537 | 471 | 421 |
|  | Newbler 100/98 | 3229 | 1298 | 489 |
|  | Newbler 60/95 | 8818 | 2508 | 571 |
|  | Velvet | 517 | 426 | 361 |
|  | Optimal | 18090 | 3604 | 699 |
| Virus-Bacteria |  |  |  |  |
|  | Celera 02 | 2499 | 1646 | 1071 |
|  | Celera 05 | 2820 | 1876 | 1117 |
|  | Celera meta | 6560 | 2804 | 1192 |
|  | Genovo | 1740 | 929 | 547 |
|  | Meta-Velvet | 547 | 445 | 364 |
|  | Minimo 100/98 | 775 | 742 | 655 |
|  | Minimo 60/95 | 669 | 518 | 428 |
|  | Newbler 100/98 | 2740 | 1130 | 475 |
|  | Newbler 60/95 | 3539 | 1325 | 511 |
|  | Velvet | 548 | 445 | 364 |
|  | Optimal | 3361 | 1333 | 571 |

Table S1 - N Statistics
