# Supplementary material for: Comparison of different assembly and annotation tools on analysis of simulated viral metagenomic communities in the gut
Source: BMC Genomics. 2014 Jan 18;15:37. doi: 10.1186/1471-2164-15-37 (PMC3901335; doi:10.1186/1471-2164-15-37)
Supplement: Additional file 2: Figure S1. — Graphical representation of the percentage of identity variation. Boxplots contrasting percentages of contig identities against their original genomes as seen in viral (A) and viral-bacterial metagenome simulations (B). Outliers are not shown. [file 1471-2164-15-37-S2.pdf]

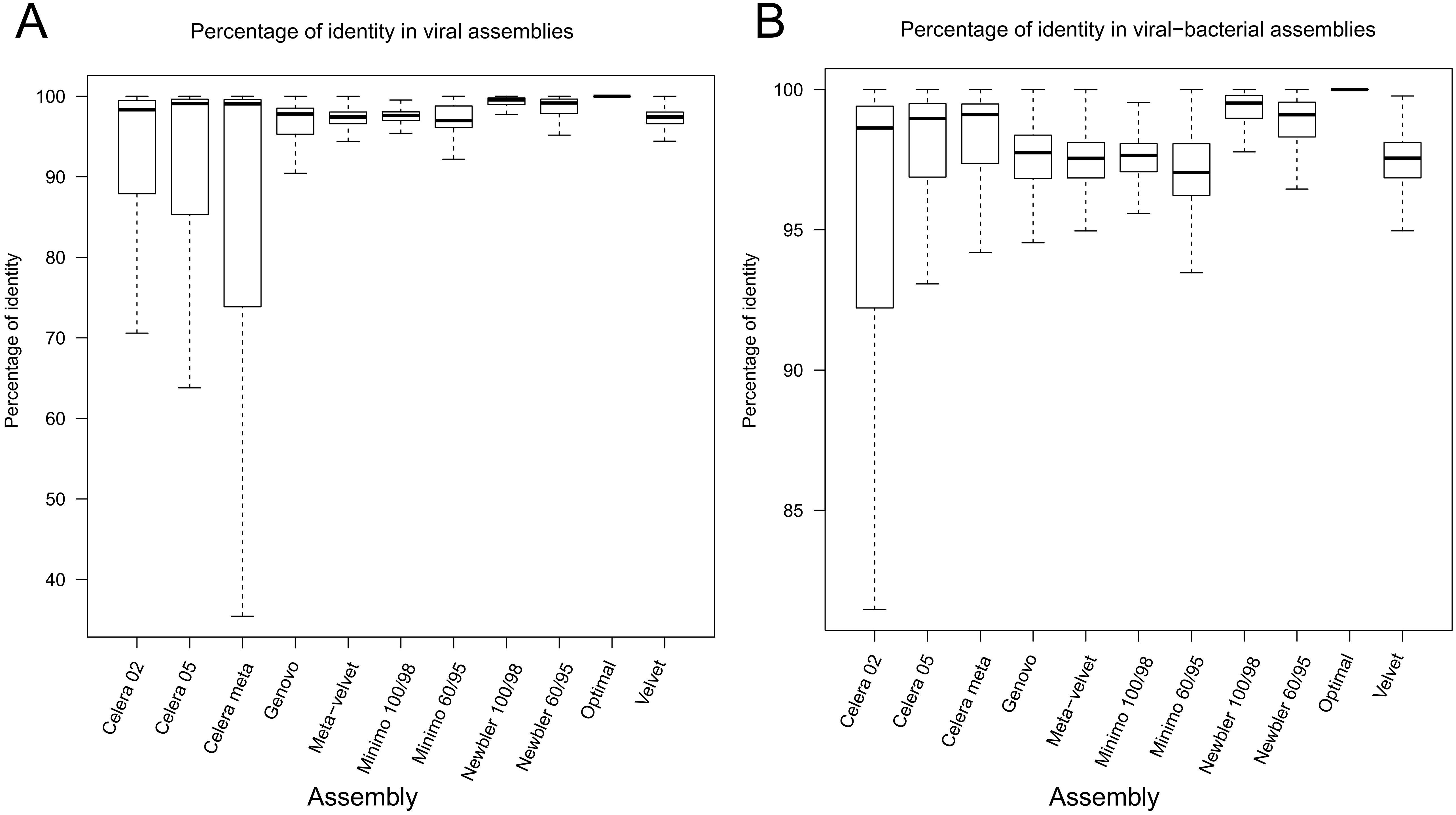
A
Percentage of identity in viral assemblies
Percentage of identity
100
90
80
70
60
50
40
Celera 02
Celera 05
Celera meta
Genovo
Meta−velvet
Minimo 100/98
Minimo 60/95
Newbler 100/98
Newbler 60/95
Optimal
Velvet
Assembly
B
Percentage of identity in viral−bacterial assemblies
Percentage of identity
100
95
90
85
Celera 02
Celera 05
Celera meta
Genovo
Meta−velvet
Minimo 100/98
Minimo 60/95
Newbler 100/98
Newbler 60/95
Optimal
Velvet
Assembly
