# Supplementary material for: Comparison of different assembly and annotation tools on analysis of simulated viral metagenomic communities in the gut
Source: BMC Genomics. 2014 Jan 18;15:37. doi: 10.1186/1471-2164-15-37 (PMC3901335; doi:10.1186/1471-2164-15-37)
Supplement: Additional file 3: Figure S2. — Hierarchical clustering from Spearman correlation coefficient. Heatmaps representing the parameter correlations in viral (A) and viral-bacterial assemblies (B). Two main clusters (green/purple) are shown between assemblies associated with their assembly statistics. Black squares indicate positive correlations and white squares negative ones. Ctgs: number of contigs, ChCtgs: percentage of chimeric contigs, GR: genomes recovered, IM: median of percentage of contig identity against its original genome, LC: Largest contig, N50, RA: percentage of reads assembled, ROG: percentage of reads assembled on their original genomes, RVB: percentage of reads within a viral-bacterial hit. [file 1471-2164-15-37-S3.pdf]

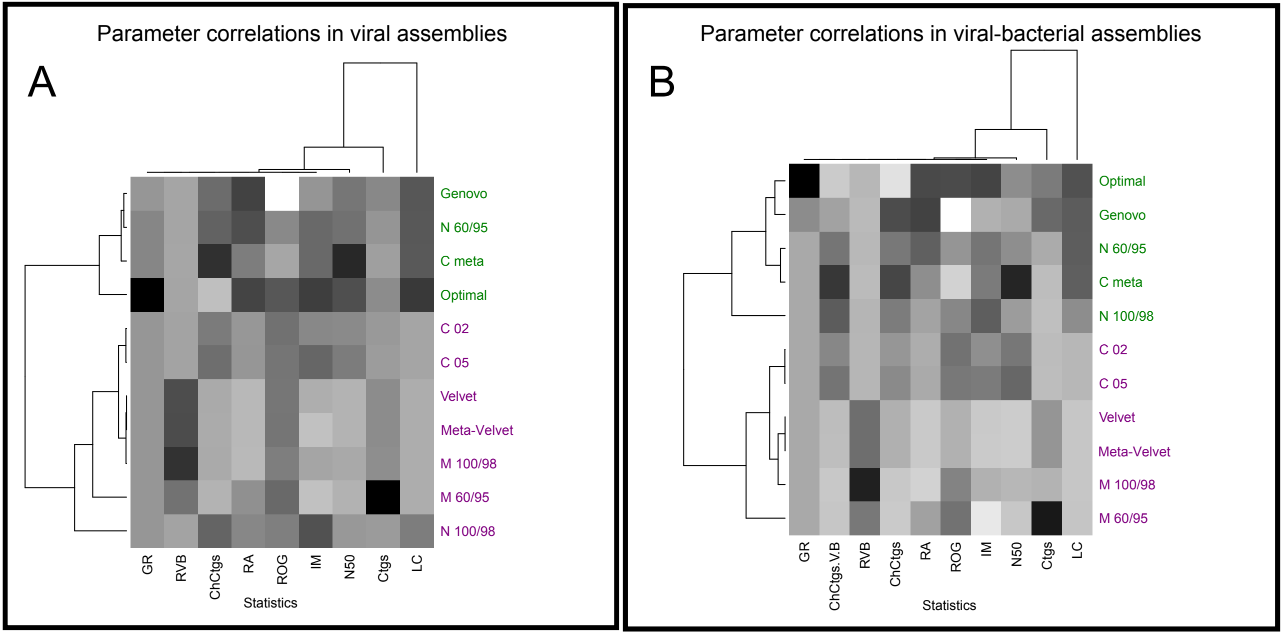
Parameter correlations in viral assemblies
A
Genovo
N 60/95
C meta
Optimal
C 02
C 05
Velvet
Meta-Velvet
M 100/98
M 60/95
N 100/98
GR
RVB
ChCtgs
RA
ROG
IM
N50
Ctgs
LC
Statistics
Parameter correlations in viral-bacterial assemblies
B
Optimal
Genovo
N 60/95
C meta
N 100/98
C 02
C 05
Velvet
Meta-Velvet
M 100/98
M 60/95
GR
ChCtgs.V.B
RVB
ChCtgs
RA
ROG
IM
N50
Ctgs
LC
Statistics
