# Supplementary material for: Comparison of different assembly and annotation tools on analysis of simulated viral metagenomic communities in the gut
Source: BMC Genomics. 2014 Jan 18;15:37. doi: 10.1186/1471-2164-15-37 (PMC3901335; doi:10.1186/1471-2164-15-37)
Supplement: Additional file 4: Figure S3. — Contig Functional Annotation. Functional annotation of the assembled reads in the viral-bacterial (A) and viral (B) metagenomes. Bar charts summarize the number of reads with correct (blue) and incorrect (red) assignations at different levels of overlapping cut off values (>10%, >30%, >50% and 100%). Dark colors are used for unassembled reads and light colors for assembled ones. Boxplots show the percentage of correct annotations (right side of A and B), considering all assemblies at four different cut offs for overlapping percentages. Boxplots per assembly (down) indicate the percentage of the correctly assembled reads for each assembly at 10 different cut off intervals (10% to 100%). [file 1471-2164-15-37-S4.pdf]

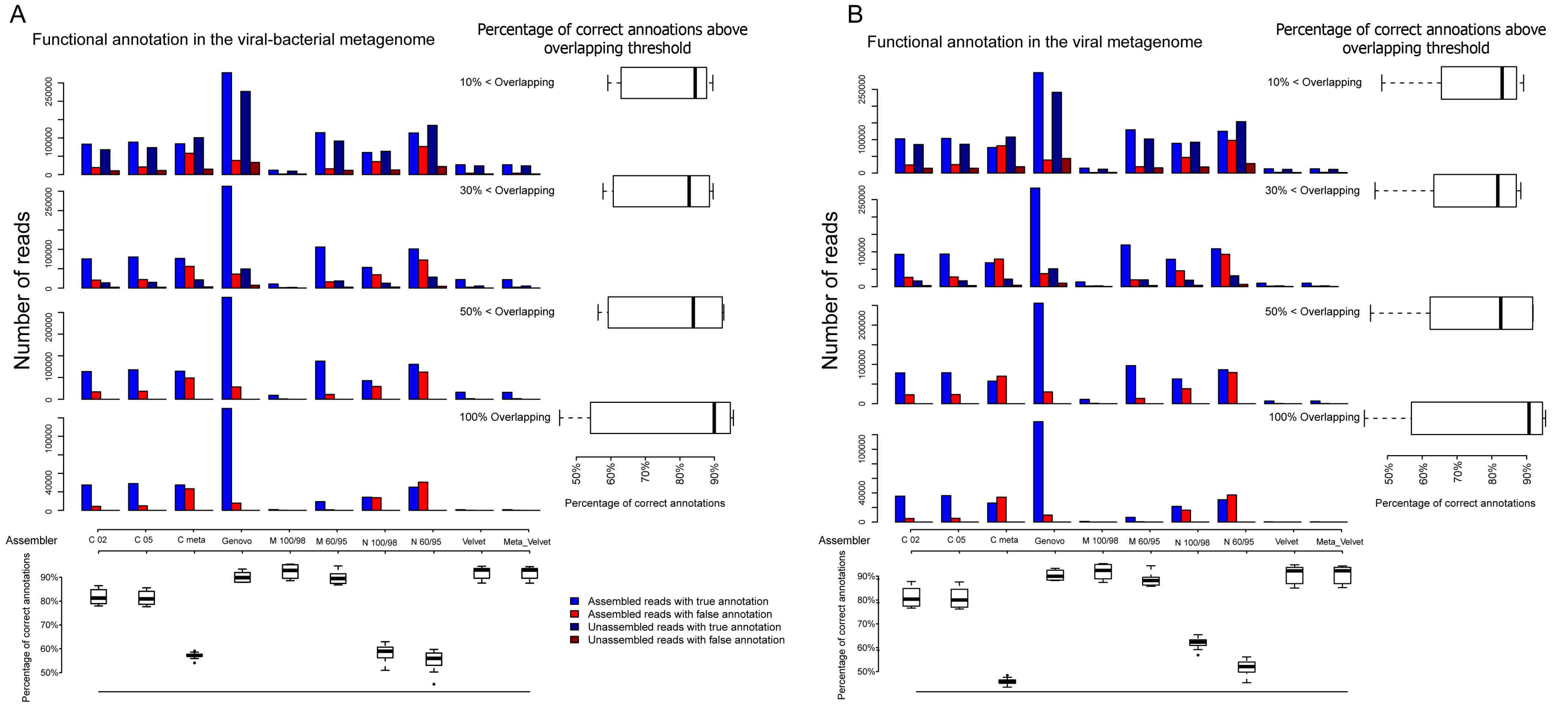
A
Functional annotation in the viral-bacterial metagenome
Percentage of correct annoations above overlapping threshold
10% < Overlapping
30% < Overlapping
50% < Overlapping
100% Overlapping
Number of reads
Percentage of correct annotations
Assembler
C 02
C 05
C meta
Genovo
M 100/98
M 60/95
N 100/98
N 60/95
Velvet
Meta_Velvet
Assembled reads with true annotation
Assembled reads with false annotation
Unassembled reads with true annotation
Unassembled reads with false annotation
B
Functional annotation in the viral metagenome
Percentage of correct annoations above overlapping threshold
Number of reads
Percentage of correct annotations
Assembler
