# Supplementary material for: Comparison of different assembly and annotation tools on analysis of simulated viral metagenomic communities in the gut
Source: BMC Genomics. 2014 Jan 18;15:37. doi: 10.1186/1471-2164-15-37 (PMC3901335; doi:10.1186/1471-2164-15-37)
Supplement: Additional file 5: Table S2. — Sensitivity and Specificity Statistics. True positives, false positives, true negatives and false negatives were calculated based on the results of taxonomic classification trials using the three custom databases Species-excluded, Genera-excluded and Families-excluded at the available taxonomic levels. The % Correct Annotation column was calculated as the sum of the true positives and true negatives. The % Incorrect Annotations is calculated as the sum of the false positives and false negatives. [file 1471-2164-15-37-S5.docx]

| Analysis | Level | TP | FP | FN | TN | Sensitivity | Specificity | % Correct Annotations | % Incorrect Annotations |
| --- | --- | --- | --- | --- | --- | --- | --- | --- | --- |
| K-mer Species-excluded | Genus | 10 | 46 | 8 | 136 | 0.56 | 0.75 | 73 | 27 |
| K-mer Species-excluded | Family | 14 | 42 | 15 | 129 | 0.48 | 0.75 | 71.5 | 28.5 |
| K-mer Species-excluded | Order | 15 | 41 | 21 | 123 | 0.42 | 0.75 | 69 | 31 |
| K-mer Genera-excluded | Family | 7 | 47 | 9 | 137 | 0.44 | 0.74 | 72 | 28 |
| K-mer Genera-excluded | Order | 8 | 46 | 15 | 131 | 0.35 | 0.74 | 69.5 | 30.5 |
| K-mer Family-excluded | Order | 1 | 39 | 3 | 157 | 0.25 | 0.8 | 79 | 21 |
|  |  |  |  |  |  |  |  |  |  |
| PhymmBL Species-excluded | Genera | 74 | 40 | 17 | 69 | 0.81 | 0.63 | 71.5 | 28.5 |
| PhymmBL Species-excluded | Family | 91 | 23 | 19 | 67 | 0.83 | 0.74 | 79 | 21 |
| PhymmBL Species-excluded | Order | 96 | 18 | 22 | 64 | 0.81 | 0.78 | 80 | 20 |
| PhymmBL Genera-excluded | Family | 24 | 39 | 19 | 118 | 0.56 | 0.75 | 71 | 29 |
| PhymmBL Genera-excluded | Order | 30 | 33 | 26 | 111 | 0.54 | 0.77 | 70.5 | 29.5 |
| PhymmBL Family-excluded | Order | 6 | 45 | 7 | 142 | 0.46 | 0.76 | 74 | 26 |
|  |  |  |  |  |  |  |  |  |  |
| tBLASTx Species-excluded | Genera | 87 | 25 | 5 | 83 | 0.95 | 0.77 | 85 | 15 |
| tBLASTx Species-excluded | Family | 99 | 13 | 8 | 80 | 0.93 | 0.86 | 89.5 | 10.5 |
| tBLASTx Species-excluded | Order | 102 | 10 | 15 | 73 | 0.87 | 0.88 | 87.5 | 12.5 |
| tBLASTx Genera-excluded | Family | 37 | 19 | 12 | 132 | 0.76 | 0.87 | 84.5 | 15.5 |
| tBLASTx Genera-excluded | Order | 40 | 16 | 20 | 124 | 0.67 | 0.89 | 82 | 18 |
| tBLASTx Family-excluded | Order | 2 | 13 | 7 | 178 | 0.22 | 0.93 | 90 | 10 |

Table S2 - Additional file 5 Table S2 - Sensitivity and specificity statistics.
