# Supplementary material for: Comparison of different assembly and annotation tools on analysis of simulated viral metagenomic communities in the gut
Source: BMC Genomics. 2014 Jan 18;15:37. doi: 10.1186/1471-2164-15-37 (PMC3901335; doi:10.1186/1471-2164-15-37)
Supplement: Additional file 6: Figure S4. — PhymmBL model and alignment method comparison. Sensitivity and specificity were compared at the genus (A), family (B) and order (C) taxonomic levels. Different iterations selected all available permutations using single models (Sm) or mixed models (Mm) and alignment using BLASTn (Bn) or tBLASTx (tBx) for the Species-excluded database. [file 1471-2164-15-37-S6.pdf]

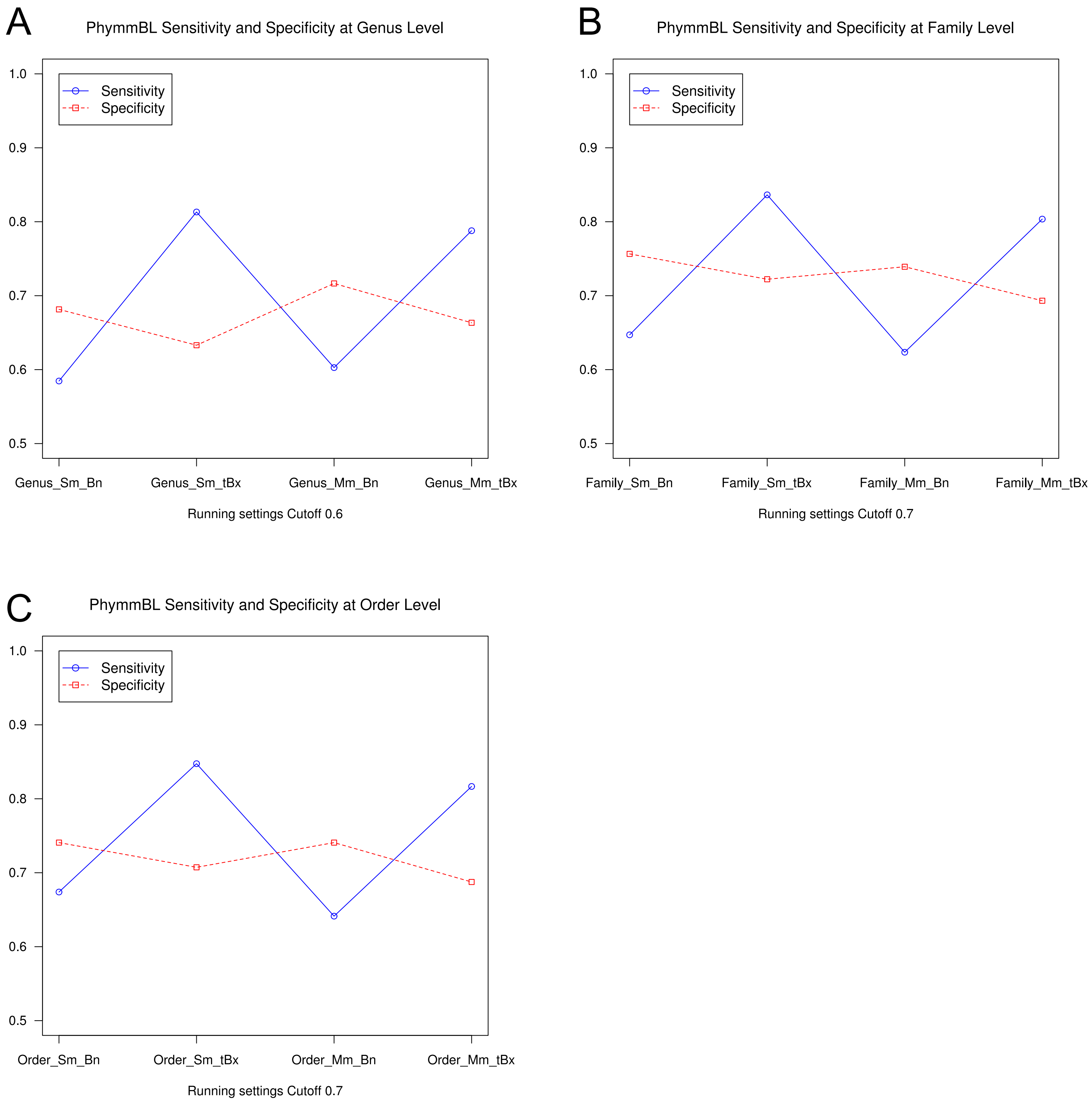
A
PhymmBL Sensitivity and Specificity at Genus Level
Sensitivity
Specificity
1.0
0.9
0.8
0.7
0.6
0.5
Genus_Sm_Bn
Genus_Sm_tBx
Genus_Mm_Bn
Genus_Mm_tBx
Running settings Cutoff 0.6
B
PhymmBL Sensitivity and Specificity at Family Level
Sensitivity
Specificity
1.0
0.9
0.8
0.7
0.6
0.5
Family_Sm_Bn
Family_Sm_tBx
Family_Mm_Bn
Family_Mm_tBx
Running settings Cutoff 0.7
C
PhymmBL Sensitivity and Specificity at Order Level
Sensitivity
Specificity
1.0
0.9
0.8
0.7
0.6
0.5
Order_Sm_Bn
Order_Sm_tBx
Order_Mm_Bn
Order_Mm_tBx
Running settings Cutoff 0.7
